# A Review of Electromagnetic Fields in Cellular Interactions and Cacao Bean Fermentation

**DOI:** 10.3390/foods13193058

**Published:** 2024-09-26

**Authors:** Tania María Guzmán-Armenteros, Jenny Ruales, Luis Ramos-Guerrero

**Affiliations:** 1 Departamento de Ciencia de Alimentos y Biotecnología, Facultad de Ingeniería Química y Agroindustria, Escuela Politécnica Nacional (EPN), Quito 170525, Ecuador; tania.guzman@epn.edu.ec (T.M.G.-A.); jenny.ruales@epn.edu.ec (J.R.); 2 Facultad de Ingeniería Mecánica y Ciencias de la Producción, Carrera de Ingeniería en Alimentos, Escuela Superior Politécnica del Litoral, Campus Gustavo Galindo, km 30.5 Vía Perimetral, Guayaquil 090902, Ecuador; 3 Grupo de Investigación Bio-Quimioinformática, Carrera de Ingeniería Agroindustrial, Facultad de Ingeniería y Ciencias Aplicadas, Universidad de Las Américas (UDLA), Quito 170503, Ecuador

**Keywords:** magnetic fields, magnetosensitivity, epigenetic modifications, cocoa bean fermentation

## Abstract

The influence of magnetic fields on biological systems, including fermentation processes and cocoa bean fermentation, is an area of study that is under development. Mechanisms, such as magnetosensitivity, protein conformational changes, changes to cellular biophysical properties, ROS production, regulation of gene expression, and epigenetic modifications, have been identified to explain how magnetic fields affect microorganisms and cellular processes. These mechanisms can alter enzyme activity, protein stability, cell signaling, intercellular communication, and oxidative stress. In cacao fermentation, electromagnetic fields offer a potential means to enhance the sensory attributes of chocolate by modulating microbial metabolism and optimizing flavor and aroma development. This area of study offers possibilities for innovation and the creation of premium food products. In this review, these aspects will be explored systematically and illustratively.

## 1. Introduction

Recently, there has been substantial progress in understanding the connection between electromagnetic fields (EMFs) and biological systems, with a particular emphasis on biotechnological processes [1,2,3,4,5,6,7]. Electromagnetic fields are defined as physical fields produced by electrically charged particles, which can influence biological entities through both direct and indirect mechanisms. The influence of magnetic fields on various biological entities has been well established, prompting researchers to adopt a dual perspective to gain a comprehensive understanding of this phenomenon. On the one hand, studies have focused on the direct effects of EMFs on biological entities, investigating the molecular and cellular mechanisms underlying their response and adaptation to these fields [6,7,8]. On the other hand, researchers have recognized the importance of considering the broader environmental context in which these processes occur, including the complex interactions between magnetic fields, microorganisms, and their surrounding ecosystems [1,2,3,4].

Studies investigating the direct effects of electromagnetic fields (EMFs) on biological systems have demonstrated that magnetic fields can modulate various cellular processes, including gene expression, protein synthesis, metabolic pathways, and signaling cascades [9,10,11,12,13]. In biotechnological applications, these interactions have been shown to enhance microbial growth and metabolic activity, thereby optimizing the production of critical bioactive compounds. Specifically, during fermentation, exposure to EMFs holds the potential to improve the biochemical composition and sensory characteristics of products such as cocoa, leading to enhanced flavor profiles and overall product quality [9,10,11,12,13].

Within the intricate web of interactions in a fermentation medium, the effects of magnetic fields become evident as they become intertwined with the complex dynamics of microbial metabolism, nutrient availability, and ecological factors. Understanding the interaction between magnetic fields and their environment is crucial for a holistic view of how microorganisms respond and adapt to magnetic stimuli. Furthermore, exploring the long-lasting effects of magnetic field exposure on microbial populations and examining synergistic or antagonistic interactions with environmental factors promises novel insights for numerous fields. As interdisciplinary research advances, the use of magnetic fields is poised to revolutionize these fields, fostering innovation and sustainable practices. In this regard, the present study aimed to contribute to systemizing the knowledge of both the effects of electromagnetic fields on biological systems and more specifically in the cacao fermentation process.

## 2. Materials and Methods

This literature review employed a multidisciplinary approach to explore the interactions between magnetic fields and biological systems, integrating both traditional research methods and advanced artificial intelligence (AI) tools. The process began with the use of the Consensus AI platform, which was instrumental in efficiently identifying and summarizing relevant scholarly articles [14]. This AI-powered tool facilitated a comprehensive search across major academic databases, including PubMed, Scopus, and Google Scholar, focusing on literature published between 2000 and 2024. The selected time frame was chosen to encompass both seminal studies and recent advances in the field.

Consensus AI initially identified approximately 300 potential articles. The AI tool provided summaries and relevance scores, which were crucial in prioritizing studies for further examination [14]. To ensure the inclusion of relevant and high-quality literature, each article underwent a digital assessment process, which involved a detailed review of the abstract, methodologies, and results to filter out non-relevant or lower-quality research. This process led to the selection of around 150 articles for further review, of which, 98 articles were retained after further assessment. In some cases, studies before the mentioned period were considered because of the need for primary information. These articles together ensured that we had highly representative information on the current state of knowledge on the topic.

In addition to the textual analysis, two figures were created to visually represent the key concepts discussed in the essay. The creation of these figures involved the use of AI tools available in Canvas, including the Magic Content feature, which facilitated the generation of visual content based on textual input [15]. This stage required the conceptualization of the necessary visual elements, focusing on the effective representation of the complex interactions between magnetic fields and biological systems at both the cellular and organismal levels.

Further refinement of these visual elements was achieved using Microsoft Bing Image Creator [16]. This AI-assisted tool enabled the production of detailed images based on accurate textual descriptions, ensuring that the scientific aspects of the interactions were accurately and engagingly represented. The final images were integrated into the figures, striking a balance between scientific rigor and visual appeal.

Key biological aspects, including magnetosensitivity, protein conformational changes, biophysical properties, reactive oxygen species (ROS) production, and gene expression, were selected due to their interconnected roles in mediating electromagnetic field (EMF) effects on biological systems. These elements were analyzed for their combined impact on cellular function, metabolic pathways, and system-wide processes. A particular emphasis was placed on their interactions to provide a holistic understanding of EMF-induced mechanisms in living organisms, with specific attention to spontaneous fermentative processes, such as those observed in cocoa beans.

## 3. Discussion

The discussion is organized into four relevant topics, which are described below. The first two tackle understanding the broad effects, applications, and mechanisms of EMFs related to biological systems. The next two focus on EMFs’ effects on biotechnological processes, including a specific analysis of a cocoa fermentation process.

### 3.1. Effects, Practical Applications, and Environmental Preservation of EMFs Related to Biological Systems

Emerging research has shed light on the remarkable ability of EMFs to exert both stimulating and inhibitory effects on a wide range of biological systems [17,18,19]. These effects extend beyond the realm of microorganisms and encompass diverse organisms, such as plants and even human and animal cells [17,18,19,20,21]. The impact of electromagnetic fields is particularly remarkable in the domain of plant growth and development. Research has shown that the precise application of EMFs can lead to accelerated seed germination, improved root growth, and a higher crop yield [21,22,23]. By carefully adjusting the electromagnetic stimulation parameters, scientists have effectively utilized these effects to enhance agricultural practices, promoting sustainable and efficient food production. Additionally, the focused use of electromagnetic fields has shown promising outcomes in enhancing the post-harvest preservation and quality of fruits, prolonging their shelf life and reducing spoilage [24,25].

Microorganisms, including bacteria, fungi, and microalgae, also exhibit fascinating responses to electromagnetic fields. Research has illuminated how controlled exposure to specific EMFs can modulate microbial growth rates, metabolic activity, and enzymatic processes [2,3,4,5,6,8,13,26]. This newfound understanding has paved the way for novel applications in biotechnology and industrial processes. For instance, electromagnetic field-mediated stimulation of microbial metabolism has been harnessed to optimize the production of valuable metabolites such as enzymes, biofuels, and pharmaceutical compounds [2,3,4,5,27,28,29]. Furthermore, the utilization of electromagnetic fields has revolutionized the process of remediating contaminated environments. Through harnessing the natural abilities of microorganisms, electromagnetic field-assisted bioremediation methods effectively break down pollutants in soil, water, and air, providing a sustainable approach to environmental protection [29,30,31,32,33]. Table 1 offers a summary of the effects and practical applications of electromagnetic fields, displaying their potential across various fields and underscoring the importance of responsible and evidence-based implementation.

The potential of electromagnetic fields extends beyond the organic and cellular levels, encompassing various practical applications in production processes, environmental preservation, and health [29,30,39]. In the field of medical therapy, electromagnetic fields have shown promise in promoting tissue regeneration, accelerating wound healing, and relieving pain and inflammation [38,39]. Non-invasive and targeted electromagnetic field-based therapies offer an alternative to traditional treatment modalities, providing patients with enhanced comfort and improved outcomes.

While the potential benefits of electromagnetic fields are substantial, it is crucial to ensure their responsible and controlled application, safeguard natural integrity, and minimize any potential adverse effects. Despite substantial advances in this field, there remains a notable absence of specific, universally standardized ranges of electromagnetic fields capable of causing measurable effects in biological systems. This gap between empirical progress highlights a complex interplay of factors contributing to the current situation. Achieving a universally applicable EMF standard requires a level of precision and robustness that considers the variability inherent in diverse biological contexts. Table 2 provides some general examples of EMF parameters that were commonly studied in the research so far. It should be noted that the specific frequencies, densities, and exposure times used in studies can vary widely depending on the specific objectives and conditions of each experiment.

### 3.2. EMF Mechanisms in Biological Systems

Magnetic fields exert their influence on biological systems through various mechanisms. These mechanisms involve magnetosensitivity, electromagnetic induction, protein conformational changes, ROS (reactive oxygen species) production, gene expression and epigenetic modifications, cellular biophysical properties, and neural activity (Table 3). Understanding these diverse mechanisms is crucial for unraveling the complex interactions between magnetic fields and biological systems and exploring their potential applications in various fields, including biomedicine, neuroscience, and biotechnology. 

#### 3.2.1. Magnetosensitivity

**Figure 1:**
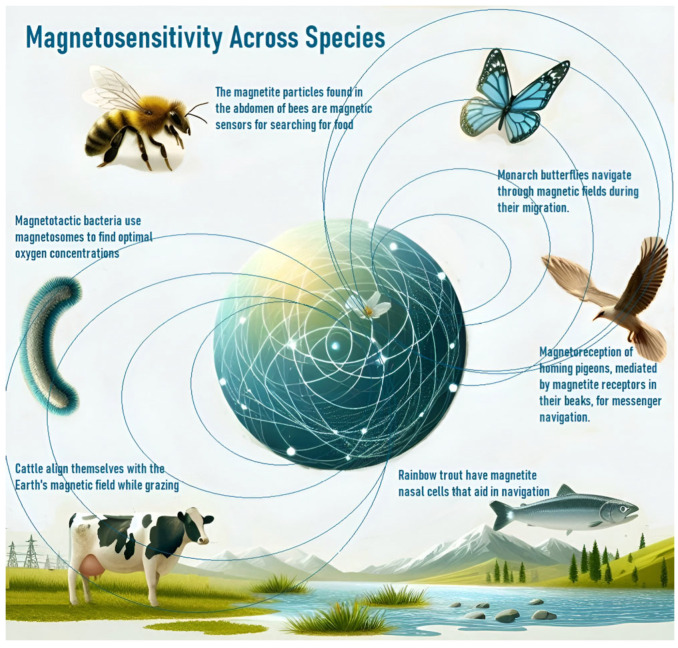
EMF-mediated orientation in various organisms.

The observation of magnetosensitivity in various organisms provides insight into the impact of magnetic fields on biological systems (Figure 1). Organisms with magnetosensitive structures, such as magnetite crystals or protein complexes, detect and respond to magnetic fields, allowing them to utilize the Earth’s magnetic field for navigation and other biological functions [43,44,45,46,47]. Recent research has highlighted the role of magnetite-based sensors in aligning crystals under a magnetic influence to generate navigational signals and noted that this ability extends beyond migratory species to include bacteria, insects, and mammals [43,44,45,46,47]. Magnetosensitivity affects behaviors such as predator avoidance and influences processes like neuronal migration and cancer therapy [48,49,50,51]. Studies have also explored the involvement of magneto-receptive proteins (like cytochromes, which may change conformation in response to magnetic fields, activating cellular pathways) and the role of ion channels and magnetosensitive bacteria in symbiotic relationships [8,9,11,12,13,52,53,54].

Magnetic fields can induce conformational alterations in proteins, particularly through interactions with paramagnetic centers like metal ions or organic radicals [55,56]. These interactions influence protein folding, enzymatic activity, and metabolic pathways by modifying electron spin configurations and protein structures [57]. Additionally, magnetic fields can impact ligand binding, altering the architecture of binding sites and affecting processes like signal transduction and gene regulation [58,59,60,61]. This knowledge could contribute to applications in biotechnology and drug design, offering opportunities to enhance enzyme activity, stabilize proteins under stress, or engineer novel ligand-binding sites [55,56,57,58,59,60,61].

#### 3.2.2. Cellular Biophysical Properties

Magnetic fields exert a profound influence on biological systems, affecting the biophysical properties of cells by modulating membrane fluidity, ion channel activities, and cytoskeleton dynamics, which in turn shape cellular behavior and function [11,12,61,62,63,64,65,66]. Studies have demonstrated that magnetic fields alter membrane organization, influencing its fluidity and permeability, and impacting processes like membrane protein function and molecular transport [11,64]. Additionally, magnetic fields affect ion channel activities, which are crucial for cellular communication and signaling, potentially modifying ion fluxes and intracellular ion concentrations [52]. These findings highlight the complex interplay between magnetic fields and cellular physiology, suggesting significant potential applications of magnetic fields in biomedicine and biotechnology.

Moreover, magnetic fields impact the cytoskeleton, a network of filaments essential for cellular structure, shape, and mechanical properties [65,66,67]. Interactions between magnetic fields and cytoskeletal elements, such as microtubules and actin filaments, can lead to changes in their organization and dynamics, influencing cellular morphology, intracellular transport, and processes like migration and gene expression [65,66,67]. This growing field of research into how magnetic fields affect biophysical properties could contribute to advancements in regenerative medicine, tissue engineering, and novel therapeutic approaches, with magnetic fields emerging as key factors influencing cellular behavior through mechanisms involving membrane dynamics, ion channels, and cytoskeleton alterations.

#### 3.2.3. Reactive Oxygen Species (ROS)

The generation of reactive oxygen species (ROS), including superoxide radicals, hydrogen peroxide, and hydroxyl radicals, in response to EMFs highlights the intricate interaction between electromagnetic fields (EMFs) and cellular redox mechanisms. ROS are essential signaling molecules and mediators of oxidative stress, and the research has indicated that magnetic fields can modulate ROS production by influencing electron transfer processes and enzymatic activities within cells, such as those involving NADPH oxidase and superoxide dismutase [68,69,70,71]. This modulation affects electron transport chain dynamics, leading to changes in ROS levels, which in turn influence key physiological processes like immune responses, cell proliferation, differentiation, and apoptosis [72,73,74,75]. Disruptions in ROS levels can result in oxidative stress, with excessive ROS causing cellular damage linked to neurodegenerative diseases, cancer, and aging, while insufficient ROS levels disrupt normal redox signaling [72,73,74,75,76,77].

Further investigation into how magnetic fields regulate ROS production, including the molecular pathways, cellular targets, and dose- and time-dependent effects, is crucial for understanding the broader impact of EMFs on cellular health. Such knowledge will deepen our understanding of redox biology and its applications in biomedicine, offering new avenues for therapeutic interventions and mitigating the negative effects associated with oxidative stress. This growing knowledge could contribute to advancements in both cellular health and biotechnology [72,73,74,75,76,77].

#### 3.2.4. Gene Expression and Epigenetic Modifications

Research has revealed that magnetic fields can modulate gene expression and contribute to epigenetic modifications, influencing cellular behavior and long-term outcomes. Several studies have shown that electromagnetic fields (EMFs) can affect gene expression by interacting with intracellular signaling pathways, transcription factors, and regulatory elements within the genome, impacting processes like cellular growth, differentiation, metabolism, and immune responses [13,78,79,80,81]. Additionally, magnetic fields are linked to epigenetic modifications such as histone changes and DNA methylation, which play crucial roles in regulating gene expression patterns and maintaining cellular identity [79,80,82,83,84]. Emerging evidence suggests that these fields may induce lasting changes in gene expression profiles and affect cellular development and differentiation [80,82,83,84].

Understanding how magnetic fields influence gene expression and epigenetic modifications remains a key area of research, focusing on signaling pathways, transcriptional regulators, and epigenetic modifiers [79,80,82,83,84]. Moreover, the potential applications of this knowledge span various fields, including biomedicine, where it could lead to therapeutic advances like targeted gene regulation and gene therapy. In agriculture, magnetic fields might enhance crop traits, stress tolerance, and yield, while in environmental science, epigenetic reprogramming could aid in contamination cleanup and ecological restoration. Continued research is essential to fully understand the molecular mechanisms and context-dependent effects of EMFs, enabling transformative advancements across these disciplines.

**Table 3:** EMFs’ interaction mechanisms with biological systems. Every mechanism illustrates the diverse impacts of magnetic fields on biological systems, highlighting their importance across various fields of research and applications. The table offers a succinct summary of each mechanism and its practical implications.

Mechanism	Description	Examples and Implications	References
Magnetosensitivity	Organisms are equipped with magnetosensitive structures that enable them to sense and react to magnetic fields. These structures enable orientation and navigation within magnetic fields and the detection of magnetic field changes.	Magnetite crystals or protein complexes act as magnetic sensors.	[85]
		Organisms can orient themselves within magnetic fields and detect changes in magnetic fields.	[45,46,47]
Changes in Cellular Biophysical Properties	Magnetic fields induce electric currents in tissues and conductive fluids, influencing cellular processes. Induced currents affect ion transport, cell signaling, and the regulation of physiological processes. Localized heat generation influences metabolic activities and cellular functions.	Magnetic fields influence ion transport across cell membranes.	[64]
		They affect cell signaling and physiological regulation.	[11,12]
		They generate localized heat within tissues.	[86]
Changes in Protein Conformation	Magnetic fields cause conformational changes in proteins, leading to modifications in their structure and function. The interaction between magnetic fields and paramagnetic centers in proteins leads to modifications in enzyme activity, ligand binding, and protein stability.	They alter enzyme activity, ligand binding, and protein stability. They impact metabolic pathways, signal transduction, and gene expression.	[61,62,63]
Reactive Oxygen Species (ROS) Production	Biological systems experience modulation of ROS generation due to magnetic fields. ROS plays a pivotal role in cell signaling and oxidative stress. Magnetic fields influence the electron transfer processes and enzymatic activities responsible for ROS generation and elimination.	Magnetic fields modulate the production of ROS molecules. They influence cellular homeostasis and physiological processes.	[69,70,71]
Gene Expression and Epigenetic Modifications	Magnetic fields influence gene expression and epigenetic modifications, regulating cellular functions. They modulate the expression of specific genes, leading to changes in cellular functions and phenotypic traits. Magnetic fields also impact epigenetic modifications to DNA.	They modulate gene expression patterns and cellular functions.	[78,79,81]
		They lead to long-term changes in cell behavior and phenotypic traits.	[87]

### 3.3. Electromagnetic Field Interactions in Biotechnological Processes

Researchers are expanding their research scope to investigate unexplored dimensions of electromagnetic fields (EMFs) in bioprocesses [88]. While previous studies have primarily examined the direct effects of EMFs and environmental factors, there is growing recognition of the need to further explore these areas. Current research is focused on the short- and long-term effects, dynamic responses and adaptations of microorganisms, and the sustained impact of EMF exposure on microbial populations within fermentation systems [1,2,3,4].

#### 3.3.1. Bacterial and Microalgae Processes

Research into the impact of static magnetic fields (SMFs) on bacteria has revealed important findings, as demonstrated by three different studies [3,13,32]. Variations in magnetic field intensity (0 mT, 50 mT, 100 mT, and 200 mT) were found to affect both the growth dynamics and the degradation rate of benzo(a)pyrene (BaP) by *Microbacterium maritypicum* [32]. For this research, cylindrical coils were used to generate a static magnetic field, with controlled temperature and magnetic induction maintained at different intensities [32]. The growth of the bacteria and their biodegradation capacity showed a direct correlation with the intensity of the magnetic field and microbial activity. Similarly, hydrogen production by *Clostridium pasturianum* was optimized at an intensity of 3.2 mT, suggesting a favorable interaction between magnetic fields and bacterial metabolism for energy production [3]. Likewise, exposure of an *Escherichia coli* mutant to between 55 mT and 2.8 Hz improved the expression of recombinant human CD22 protein that regulates the cellular response to different pathogens [13].

Research on the effects of magnetic fields on microalgae has consistently highlighted how magnetic fields affect biochemical pathways and gene expression, ultimately influencing the production of essential biochemical compounds in microalgae. Studies on *Nannochloropsis gaditana*, *Scenedesmus obliquus*, *Chlorella sorokiniana*, *Chlorella pyrenoidosa*, and *Tetraselmis obliquus* indicated that exposure to static magnetic fields modulates essential cellular processes [6,26]. These findings highlight the changes in the biochemical composition [6] and metabolic profiles [7] of these microalgae under the influence of a magnetic field [6,26].

#### 3.3.2. Fungal and Yeast Processes

Different studies have shown that exposure of fungal strains to magnetic fields can cause increases in biomass, bioproducts, enzymatic activity, and biodegradation processes. These findings provide valuable information on the potential applications of magnetic field stimulation in bioprocesses for wastewater treatment, enzyme production, and fermentative processes [2,19,33]. The effects of extremely low frequency (ELF) static magnetic fields (SMFs) on bioethanol production by *Saccharomyces cerevisiae* in bioreactors showed an increase of 33% in bioethanol production [2]. This phenomenon was attributed to the stimulation of plasma membrane H+-ATPase activity, which improved yeast metabolism during fermentation.

The action of a static magnetic field on the biodegradation of contaminants by *Candida tropicalis* showed higher removal percentages compared to other reactors [33]. In this research, notable improvements in the biodegradation of azo dyes by the yeast *Candida tropicalis* were achieved under hypersaline conditions using sequential batch reactors. The results indicated that magnetic fields increased the biomass multiplication, enzymatic activity, decolorization efficiency, and chemical oxygen demand (COD). A microbial community analysis revealed changes in bacterial and fungal populations in response to the treatments, which showed promising results for improving the efficiency of biodegradation processes [33]. This study presents a potentially applicable method for the purification of hypersaline industrial wastewater, highlighting the potential of magnetic fields in enhancing key biotechnological processes.

In the production of β-fructofuranosidases by *Aspergillus tamarii* under the influence of a static magnetic field, high stability of the enzymes at 50 °C was demonstrated [19]. Furthermore, the enzyme activity was positively influenced by the magnetic field, increasing its affinity for the substrate. This research provides valuable information on the impact of magnetic fields on enzyme activity, highlighting the improvements in enzyme kinetic parameters [19]. Exposure of *Cunninghamella echinulata* to high-intensity magnetic fields caused spore mutation, giving rise to mutants with a high lipid content [81]. Among these, Mutant-29 exhibited the highest yield of gamma-linolenic acid (GLA) and total lipid content. The optimal conditions for this increased production included glucose as a carbon source, nitrate as a nitrogen source, and a low potassium content [81]. This study demonstrated that magnetic field-induced mutation can significantly enhance polyunsaturated fatty acid (PUFA) production in fungi.

### 3.4. Influence of EMFs on Cocoa Fermentation Process

**Figure 2:**
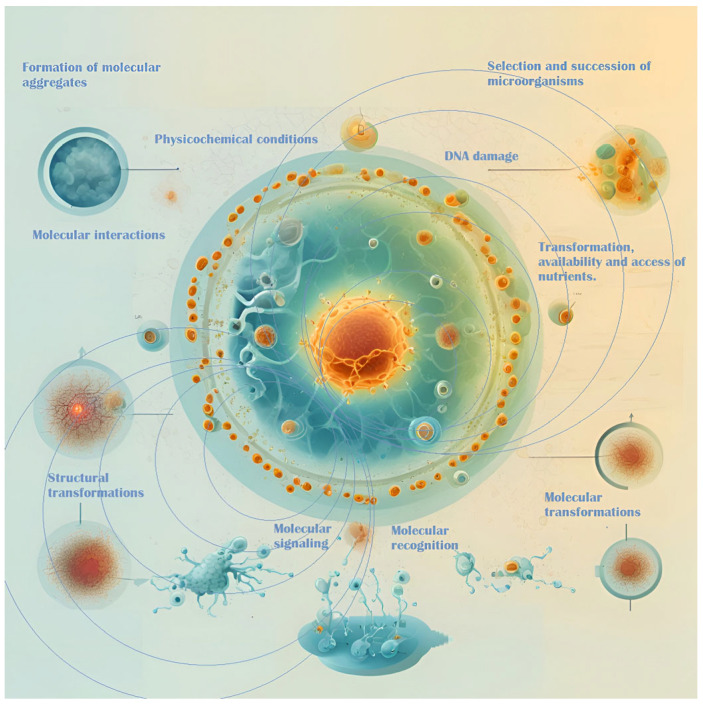
EMFs’ interactions with microbes and their surrounding environment.

Microorganisms respond to magnetic fields through complex communication mechanisms, including quorum sensing and intercellular signaling, which regulate their physiological and metabolic processes [11,12,89]. These mechanisms are essential for microbial interactions, community dynamics, and the overall stability of fermentation within the complex ecosystem of the fermentation medium (Figure 2). Magnetic fields can influence the selection and succession of microbial species, thereby shaping the microbial consortia involved in fermentation [90]. Gaining insight into these interactions offers the potential for optimizing fermentation processes, enhancing product quality, and improving sustainability, particularly in applications such as cocoa fermentation [90,91].

Cocoa bean fermentation represents a valuable model for studying interactions between microorganisms and the complex dynamics of their communities. Due to its well-defined and reproducible nature, this system allows us to investigate the relationships between microorganisms and their environment in detail [92,93,94]. By analyzing the mechanisms that regulate microbial colonization, metabolic activities, and the resulting physical changes in cocoa beans, it is possible to extrapolate these findings to other fermentation systems. This provides a more complete understanding of the dynamics of microbial ecosystems and their impact on the quality of the final product.

The cocoa bean fermentation process involves a wide range of microbial species, which function as a unique interconnected ecosystem that depends on several environmental factors that are crucial for their successful progression [92,93,94]. Factors such as oxygen tension, temperature, humidity, and pH influence the fermentation environment during cocoa pulp fermentation. The dynamic interaction between these factors is regulated by the metabolic activity of the microbial species involved [90,92,93,94].

The complexities of microbial metabolism and the physical changes induced by fermentation of cocoa beans have wide-ranging implications. In the field of food science, they can inform strategies for optimizing fermentation processes, improving product consistency, and enhancing the development of desirable sensory attributes in cocoa-based products [90,95]. The cocoa bean ecosystem’s native microorganisms undergo a natural selection process, gradually colonizing the pulp according to their distinct metabolic capabilities [90,92,93,94]. This selective colonization results in the establishment of a specialized microbial consortium that exhibits synergistic interactions and concerted metabolic activities [93,94]. The metabolic potential of these microorganisms dictates their ability to thrive within the cocoa pulp environment, thereby contributing to the overall fermentation process [90,92]. The metabolic activities of the microorganisms involve the degradation of the organic compounds found in cocoa pulp, resulting in the generation of a plethora of metabolic byproducts that contribute to the development of distinctive flavors, aromas, and chemical transformations within the cocoa beans [90,92,93,94,95].

Electromagnetic fields (EMFs) can influence the growth and metabolic activity of the microorganisms involved in cocoa fermentation, substantially altering the dynamics of the populations during this process [90,91,96,97,98]. The main microbial groups (lactic acid bacteria (LAB), yeasts, and acetic acid bacteria (AAB)) exposed to an oscillating EMF of 5 and 80 mT experienced substantial changes. In all treatments, a constant inhibitory effect on LAB was observed. In contrast, both yeasts and AAB showed notably higher growth at intensities of 5 and 42 mT, while exposure to 80 mT resulted in the lowest concentration of the three microbial groups evaluated.

Electromagnetic fields can modify microbial growth rates [96], and the colonization of specific microbial species directly impacts the fermentation kinetics and the metabolic profiles of the generated by-products [90,96]. In this context, it is reasonable to assume that magnetic fields could alter the enzymatic profiles of cocoa-fermenting microorganisms, leading to variations in the production of key metabolites and flavor precursors. These metabolic modifications could offer opportunities for customizing sensory characteristics and improving quality attributes in cocoa-derived products [90,98]. Furthermore, magnetic fields could indirectly influence the fermentation environment, affecting the transport of nutrients and ions, which would optimize the availability of essential components for microbial growth and metabolism [90,98]. Another possible mechanism of action of magnetic fields is the modification of microbial populations and their behavior, which would have a significant impact on the cocoa fermentation process [90,91,96,97,98].

Electromagnetic fields have shown promising potential in improving the sensory quality of chocolate by optimizing certain desirable characteristics [96,97]. A recent study demonstrated that exposure to variable magnetic fields, ranging from 5 to 80 mT, induced significant sensory changes in cocoa beans, which correlated with the dynamics of the predominant microbial genera during fermentation [90]. Metagenomic analyses revealed a higher microbial diversity in treatments with low magnetic field intensities (5 mT), where the microbial genera associated with the production of favorable aroma precursors, such as *Hanseniaspora*, *Pichia*, *Lactobacillus*, and *Tatumella*, predominated. In contrast, exposure to higher-intensity magnetic fields (80 mT) favored the development of microbial species linked to undesirable sensory attributes, such as *Pseudomonas*, *Mucor*, *Klebsiella*, and *Acetobacter* [90]. These findings suggest that magnetic field intensity plays a critical role in shaping the microbial community and, consequently, the sensory profile of fermented cocoa beans. Sensory evaluations also highlighted significant improvements in the aroma, flavor, and texture of the beans subjected to low-intensity fields (5 mT) [90].

During cocoa bean fermentation, microbial metabolism induces both external and internal changes in the beans [95]. Externally, fermentation visibly alters the appearance and texture of the beans, affecting their color, size, and surface characteristics, and, in some cases, they develop specific patterns or marks [97,99,100]. Internally, biochemical transformations occur that impact the composition of the beans, such as the conversion of complex carbohydrates into simple sugars, the breakdown of proteins, the generation of volatile compounds, and the synthesis of bioactive compounds [95,96,97,99,100]. These biochemical changes are determinants in the development of flavor, aroma complexity, and other key sensory attributes of the final chocolate [90,91,95].

Magnetic fields can indirectly affect cocoa fermentation by influencing the surrounding environmental conditions such as the temperature, oxygen availability, and pH, all of which significantly impact the fermentative processes [87,92]. Under optimal experimental conditions (5 mT, 22.5 min of exposure, and 1.6% inoculum), a notable increase of 110% in fermentation yield and 120% in overall bean quality was observed compared to the control group [97]. Electromagnetic fields (EMFs) significantly impacted the variables associated with cocoa bean fermentation. The highest temperatures were recorded with the 5 and 42 mT treatments, while the 80 mT treatment resulted in the lowest temperatures [97]. The maximum pH value was reached at 5 mT, while the minimum was observed at 80 mT. The highest levels of soluble solids and moisture were also recorded with the 80 mT exposure, whereas the 5 and 42 mT treatments exhibited the lowest values for these parameters. Additionally, the weight loss and fermentation rates were significantly lower under the 80 mT treatment [97].

The effect of extremely low frequency (ELF) magnetic fields on the fermentation of unfermented dried cocoa beans was also investigated using field intensities of 100 μT, 200 μT, and 300 μT, with exposure times of 15, 45, and 75 min [98]. Although no significant changes in temperature were observed during fermentation, the drying process was accelerated, leading to a more rapid reduction in moisture content compared to the control group [97]. Furthermore, beans exposed to ELF fields exhibited significantly a higher pH level and alcohol content, with the highest alcohol content observed using 200 μT for 45 min [97]. These findings suggest that ELF magnetic fields may serve as an effective method to enhance the flavor quality of unfermented dried cocoa beans, providing a novel approach to optimizing post-harvest processes in cocoa production.

Magnetic fields have been demonstrated to affect physical properties such as surface tension, viscosity, and crystallization behavior across various systems [11,12]. In the context of cocoa fermentation, it is plausible that these fields influence the formation and stability of cocoa butter crystals, which are crucial for achieving the desired texture and mouthfeel in chocolate products.

Recent studies have shown that gamma irradiation can induce detectable changes in the sensory profile of cocoa liquor, impacting attributes such as fruitiness, bitterness, and rancidity, with the effects varying with the irradiation dose. Gamma irradiation enhanced the fruitiness and floral notes in the Nacional variety, while the CCN-51 variety exhibited increased rancidity [91]. High doses of gamma irradiation (≥1 kGy) led to elevated rancidity, which was attributed to oxidative radiolysis of unsaturated fatty acids. Conversely, low to moderate doses (<1 kGy) better preserved the desirable sensory attributes [91]. Additionally, the induction of extremely low frequency (ELF) magnetic fields during fermentation appeared to affect the composition of the cocoa liquor [91]. However, more controlled experiments are needed to fully elucidate these effects. Understanding these physical transformations is essential for improving chocolate quality.

## 4. Conclusions

The mechanisms underlying the influence of magnetic fields cover several aspects, including magnetic sensitivity, changes in protein conformation, changes in cellular biophysical properties, reactive oxygen species (ROS) production, changes in gene expression, and epigenetic modifications. These effects can vary significantly between different organisms and cellular systems, opening up new possibilities for innovative applications in biotechnology, agriculture, remediation, and especially in the fermentation process for chocolate production.

The influence of magnetic fields on biological systems is complex and multifaceted, affecting molecular structures and cellular processes at the genetic and epigenetic levels. These impacts have the potential to transform diverse areas, providing opportunities to optimize biotechnological and agricultural processes, improve remediation practices, and refine the fermentation techniques in chocolate production. Understanding and applying these mechanisms can lead to significant advances in the quality and efficiency of industrial and environmental processes.

## Figures and Tables

**Table 1:** EMF effects on different biological systems, including microorganisms, plants, fruits, human cells, and industrial production.

Biological System	Effects and Applications	References
Microorganisms	Modulation of microbial growth rates, metabolic activity, and enzymatic processes. Modulation of microbial metabolism for valuable metabolites’ production, biotechnology, and industrial processes. Electromagnetic field-assisted bioremediation techniques for efficient pollutant degradation.	[1,2,3,4,5,6,7,8,32]
Plants and Fruits	Accelerated seed germination and enhanced root growth. Increased crop yield and improved agricultural practices. Post-harvest preservation and quality enhancement of fruits. Extension of fruit shelf life and reduction in spoilage.	[23,25,34,35]
Human Cells and Therapy	Promotion of tissue regeneration and wound healing. Alleviation of pain and inflammation. Non-invasive and targeted therapies as alternatives to traditional treatment modalities.	[36,37,38]
Industrial Production	Optimization of fermentation processes for cost-effective and sustainable production of commodities. Enhancement of efficiency and yield in various industrial fermentation processes.	[1,2,3,4,5,6,7,8]
Environmental Preservation	Remediation of contaminated sites through electromagnetic field-assisted bioremediation techniques. Mitigation of the harmful effects of pollutants in soil, water, and air. Preservation of ecological integrity and natural habitats.	[30,31,32,33]

**Table 2:** General examples of commonly studied EMF parameters by category.

Category	Frequency (Hz)	Field Density (mT)	Exposure Time	Example and Reference
Plants	Range from extremely low frequency (ELF) to radiofrequency (RF) ranges. ELFs: from 1 to 300 Hz. RFs: from kHz to GHz.	The ELF or SMF fields can be used depending on the study (0.1 to around 300 mT)	From a few minutes to several days or even weeks. Short-term exposures of a few hours are common, but some studies involve longer-term exposures of 24 h or more.	Biological System: *Hordeum vulgare* L. Frequency: SMF (0 Hz) Density: 250 mT Exposure Time: 4 days [40]
Animals	Range from ELF to RF Reports: 50 Hz, 60 Hz, 900 MHz, and 2.45 GHz.	Depending on the study, from fractions of a μT to several mT.	Can vary widely based on the research objectives. Short-term exposures can be as brief as a few hours, while long-term studies might extend over several weeks or even months. Common exposure durations include 24 h, 48 h, and 7 days.	Biological System: Rat brain Frequency: ELF (50–60 Hz) Density: 4.3 or 12.9 mT Exposure Time: 21 days [41]
Human cells	Range from ELF to RF. Reports: 50 Hz, 60 Hz, 900 MHz, and 2.45 GHz.	From mT or μT for ELF fields. For radio frequency EMFs, a specific absorption rate (SAR) is used, measured in watts per kilogram (W/kg). From fractions of a μT to several mT for ELF fields. SAR values range from a few mW/kg to higher values for RF fields.	Based on research objectives and safety considerations. Short-term exposures can be as brief as a few minutes to an hour, while longer-term studies might extend over several hours. Common exposure durations include 15 min, 30 min, and 1 h for short-term studies, and up to 24 h for longer-term studies.	Biological System: Human amniotic (FL) cells Frequency: ELF 50 Hz Density: 0.2 or 0.4 mT Exposure Time: 1 or 24 h [42]
Fermentation Processes	Wide range. ELF range: around 50–60 Hz due to their potential biological effects and ease of generation.	Based on the type of fermentation process and the specific objectives of the study. For ELF fields, densities might range from a fraction of a μT to several μT.	Short-term exposures might last from minutes to hours, while longer-term studies could extend throughout the fermentation process, which can range from several hours to days or even weeks.	Biological System: Bioethanol production by *S. cerevisiae* Frequency: ELF (50–60 Hz) Density: 10 mT Exposure Time: 2 h [2]
Microorganisms	Range from ELF to RF. ELFs: 50–60 Hz. RFs: microwaves (900 MHz, 2.45 GHz)	From fractions of μT to several T for ELF fields.	From a few minutes to several hours, while longer-term studies could extend over multiple hours or even days. Common exposure durations include 15 min, 30 min, 1 h, and up to 24 h.	Biological System: Airborne fungi Frequency: ELF (50–60 Hz) Density: 5 mT Exposure Time: 2 h [29]

## Data Availability

No new data were created or analyzed in this study. Data sharing is not applicable to this article.
